# Public health round-up

**DOI:** 10.2471/BLT.21.010421

**Published:** 2021-04-01

**Oxygen shortages in the pandemic**

A nurse examines an oxygen concentrator donated by the World Health Organization in a ward of the isolation centre at Abaarso Tech University Daryeel Hospital in Hargeisa, Somalia, where there has been an increase in demand for medical oxygen during the COVID-19 pandemic. A new taskforce has been launched under the ACT-Accelerator initiative to assess oxygen demand and to secure oxygen supplies and technical support for the worst-affected countries.

**Figure:**
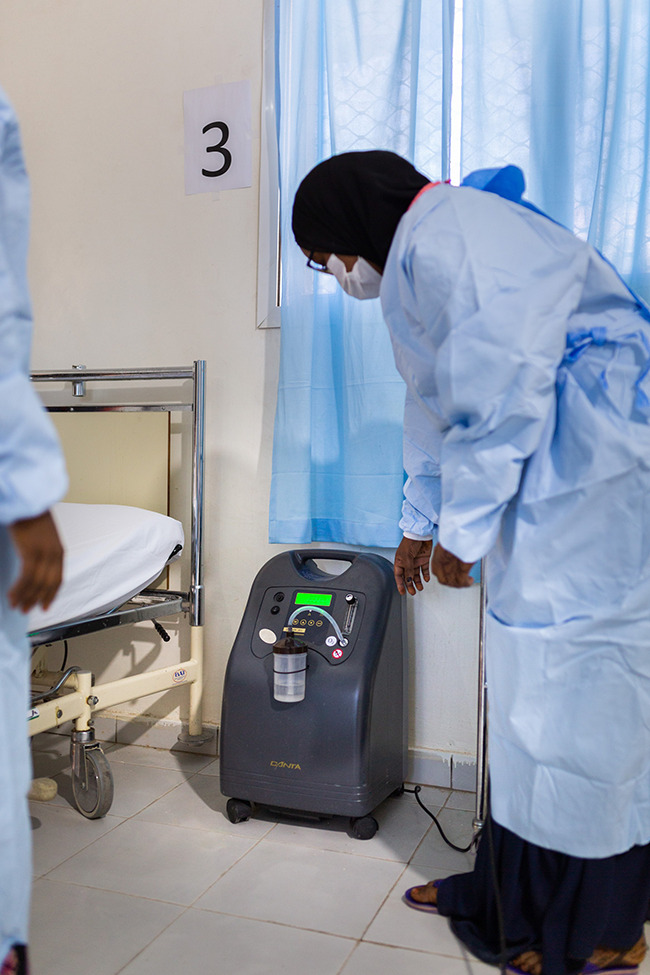
WHO/Blink Media/Mustafa Saeed

## COVAX delivering

Côte d’Ivoire and Ghana started vaccinating health workers against coronavirus disease 2019 (COVID-19) on 1 March, becoming the first countries to begin vaccination campaigns with doses supplied through COVAX, the vaccine pillar of the Access to COVID-19 Tools (ACT) Accelerator, a global collaboration to accelerate the development, production and equitable access to new COVID-19 diagnostics, therapeutics and vaccines. A further 11 million doses were delivered through COVAX in the first week of March.

http://bit.ly/3vjFMoV

## First single dose COVID-19 vaccine approved

The World Health Organization (WHO) listed the first single dose COVID-19 vaccine for emergency use in all countries. Delivered with one injection, the vaccine is stored at −20°C, but can be kept for three months at 2–8°C. The Ad26.COV2.S vaccine was listed on 12 March and was developed by Johnson & Johnson subsidiary Janssen.

“Every new, safe and effective tool against COVID-19 is another step closer to controlling the pandemic,” said WHO Director-General Tedros Adhanom Ghebreyesus. “But the hope offered by these tools will not materialize unless they are made available to all people in all countries.”

As of 12 March, the COVAX Facility had contracted to purchase 500 million doses of the vaccine.

http://bit.ly/38FU49E

## Revised ACT-Accelerator strategy

The ACT-Accelerator Strategy and Budget was revised to reflect recent developments in the COVID-19 pandemic and the tools being developed to respond. Published on 12 March, the new strategy was drafted in response to three key developments: the approval of safe and effective COVID-19 vaccines; the emergence of virus variants; and insufficient investment in global solutions to scale COVID-19 tools.

Despite generous donor contributions amounting to US$ 11.0 billion as of 12 March – including a recent commitment of US$ 4.3 billion in new funding by Canada, the European Commission, Germany, Japan and the United States of America – the ACT-Accelerator requires an additional US$ 22.1 billion in 2021 to achieve its objectives.

http://bit.ly/3cuXYmJ

http://bit.ly/3eRhfSr

## Oxygen taskforce launched

A new COVID-19 oxygen emergency taskforce was launched under the ACT-Accelerator therapeutics pillar. The launch is part of an initiative which will see the therapeutics pillar taking on a new role in coordinating and advocating for the increased supply of medical oxygen as part of the COVID-19 pandemic response.

Launched on 25 February, the taskforce is assessing oxygen demand, and working to secure oxygen supplies and technical support for worst-affected countries. COVID-19 has put huge pressure on health systems, with 25 countries reporting surges in demand.

http://bit.ly/3lgQ3gK

## Polio concerns

Circulating vaccine-derived poliovirus (cVDPV) cases rose from 378 in 2019 to 1009 in 2020, heightening concerns about its international spread. In a 19 February statement, the Emergency Committee under the International Health Regulations (2005) highlighted various factors driving the increased incidence of cVDPV, including the impact of the COVID-19 pandemic on routine immunization and polio prevention activities. The committee assessed the risk of international spread of cVDPV2 to be “very high”.

The committee also expressed concern regarding wild poliovirus, 140 cases of which were reported in Afghanistan and Pakistan in 2020 (56 in Afghanistan, 84 in Pakistan), stating that the risk of international spread of wild poliovirus “may be at the highest level since 2014”.

http://bit.ly/3tlaRqg

## Coordinating the Ebola response

Ministers of health from Guinea and neighbouring countries agreed to establish a coordination mechanism and enhance cross-border collaboration to contain an outbreak of Ebola virus disease which was declared in Guinea on 14 February.

Dr Abdou Salam Gueye, Regional Emergency Director at WHO’s Regional Office for Africa, welcomed the decision, stressing the importance of a regional approach to the outbreak in view of extensive cross-border trade and close social ties between the countries.

In support of Guinea’s health authorities, WHO and partner organizations have ramped up the response to the outbreak which includes a targeted vaccination campaign. As of 2 March, 32 000 Ebola vaccine doses had been delivered and 1317 people had been vaccinated.

As of the same date 13 people had been reported to be infected with the virus (nine confirmed, four probable) in Guinea. No cases had been reported in the surrounding countries.

http://bit.ly/38ERArT

## Violence against women

Nearly a third of women aged 15 to 49 (736 million of 2.3 billion) are subjected to physical and/or sexual violence. This is according to a new report based on a 2018 analysis of prevalence data from 2000 to 2018 across 161 countries and areas, published by WHO and partners on 9 March.

Intimate partner violence is by far the most prevalent form, affecting around 641 million women. However, 6% (95 million of 2.3 billion) report being sexually assaulted by someone other than their husband or partner. Given the high levels of stigma and underreporting of sexual abuse, the true figure is likely to be significantly higher.

“Violence against women is endemic in every country and culture, causing harm to millions of women and their families, and has been exacerbated by the COVID-19 pandemic,” said WHO Director-General Tedros Adhanom Ghebreyesus, calling for sustained efforts by governments, communities and individuals to change harmful attitudes, improve access to opportunities and services for women and girls, and foster healthy and respectful relationships.

http://bit.ly/3rPLNr4

## New breast cancer initiative

A global breast cancer initiative was launched with the goal of reducing breast cancer mortality by 2.5% per year until 2040. Launched by WHO on 9 March, the initiative reflects increased recognition of breast cancer as a public health priority and seeks to address inequities in access to breast cancer diagnosis and treatment.

Working in collaboration with other United Nations agencies and partner organizations, WHO will provide guidance to governments on how to strengthen systems for diagnosing and treating breast cancer, which in turn is expected to lead to improved capacities to manage other types of cancer.

Breast cancer has overtaken lung cancer as the world’s mostly commonly diagnosed cancer and is responsible for one in six of all cancer deaths among women, according to statistics released by the International Agency for Research on Cancer in December 2020.

http://bit.ly/3tnHFii

**Cover photo**

A woman receives a COVID-19 vaccine provided by COVAX, the vaccines pillar of the Access to COVID-19 Tools Accelerator, which began supplying doses to vaccinate health workers in Côte d’Ivoire and Ghana on 1 March 2021.

**Figure:**
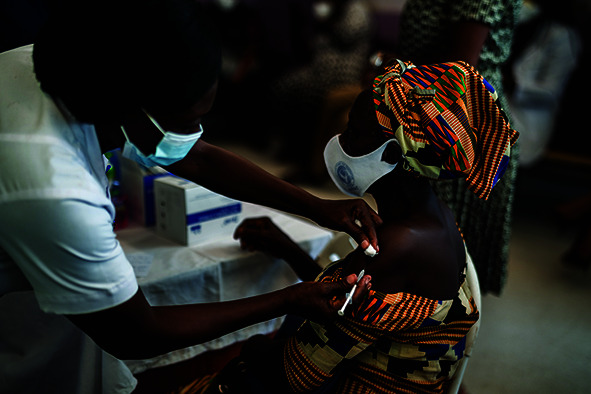
WHO/Blink Media/Nana Kofi Acquah

## First digital health guideline

WHO launched its first SMART Guideline, as part of efforts to support the development and adaptation of digital health systems. The guideline is devoted to antenatal care (ANC) and builds on groundwork done for the antenatal care recommendations toolkit which was launched by WHO and partners in June 2020.

The guideline includes a digital adaptation kit, an implementation guide for machine-readable recommendations and a WHO digital ANC module for health care providers.

The SMART Guidelines are being developed in recognition of the complexity of digital health initiatives and provide a systematic, transparent and testable structure for countries to work through. More SMART guidelines will be released later this year.

http://bit.ly/2NjFPQr

## Radiotherapy procurement

WHO and the International Atomic Energy Agency (IAEA) released new guidance on the procurement of radiotherapy equipment. Released on 5 March, the guidance is intended for medical physicists, biomedical and clinical engineers, and radiation oncologists and was developed as part of the ongoing collaboration between WHO and the IAEA to foster safety and quality in the medical use of radiation technology which is still lacking in many countries.

http://bit.ly/3cuBMcs

## World hearing report

Nearly 2.5 billion people worldwide – or roughly 1 in 4 people – will be living with some degree of hearing loss by 2050. This is according to WHO’s first *World report on hearing* which was released on 2 March. The report identifies the main obstacles to ear and hearing care access, including lack of awareness, stigmatization and the dearth of ear and hearing specialists in many countries. The report underlines the need to rapidly step up efforts to prevent and address hearing loss, notably by investing and expanding access to ear and hearing care services.

http://bit.ly/3eDKQ1x

## Consolidated malaria guidelines

WHO launched consolidated guidelines for malaria, bringing together the Organization’s most up-to-date recommendations for malaria in one easy-to-navigate online platform. The guidelines are designed to support malaria-affected countries in their efforts to reduce and, ultimately, eliminate a disease that continues to claim more than 400 000 lives each year.

http://bit.ly/38HJEX7

**Looking ahead**

7 April. World Health Day. http://bit.ly/2OXC3fN

13–22 April. Fair Pricing Forum. http://bit.ly/3eDLD2v

24–30 April. World Immunization Week. http://bit.ly/3vzVEno

